# Differential Immune Responses to New World and Old World Mammalian Arenaviruses

**DOI:** 10.3390/ijms18051040

**Published:** 2017-05-12

**Authors:** Hinh Ly

**Affiliations:** Department of Veterinary and Biomedical Sciences, University of Minnesota, Twin Cities, 1988 Fitch Ave., Ste 295, Saint Paul, MN 55108, USA; hly@umn.edu; Tel.: +1-612-625-3358

**Keywords:** arenaviruses, Lassa, Junin, hemorrhagic fever, immune evasion, vaccine

## Abstract

Some New World (NW) and Old World (OW) mammalian arenaviruses are emerging, zoonotic viruses that can cause lethal hemorrhagic fever (HF) infections in humans. While these are closely related RNA viruses, the infected hosts appear to mount different types of immune responses against them. Lassa virus (LASV) infection, for example, results in suppressed immune function in progressive disease stage, whereas patients infected with Junín virus (JUNV) develop overt pro-inflammatory cytokine production. These viruses have also evolved different molecular strategies to evade host immune recognition and activation. This paper summarizes current progress in understanding the differential immune responses to pathogenic arenaviruses and how the information can be exploited toward the development of vaccines against them.

## 1. Introduction

Arenaviruses are single-stranded ambisense RNA viruses that are divided into Old World (OW) and New World (NW) viruses based on their phylogenetic, serological, and geographical differences. Many of these mammalian viruses are carried by rodents, but can cause significant morbidity and mortality in humans. They are therefore emerging zoonotic viruses of great importance to public health. Lassa virus (LASV) and Lujo virus (LUJV) are OW viruses found in Africa, and Junín virus (JUNV) and the other NW arenaviruses found in South America (Table 1) can cause severe and sometimes fatal hemorrhagic fever (HF) infections. Prophylactic and therapeutic treatments for these pathogenic infections are currently limiting. Candid #1 is the only vaccine that was used extensively to prevent Argentine hemorrhagic fever caused by JUNV [1]. Ribavirin is the only licensed antiviral to treat HF caused by some arenaviruses, but it has mixed success and significant toxicity [2].

LASV infects approximately half million individuals and can cause up to 5000 deaths annually in several endemic countries in West Africa [3]. LASV infection can result in varied disease manifestations, ranging from non-symptomatic to multi-organ failure and death. Patients infected with LASV exhibit symptoms that include fever, malaise, petechial hemorrhage, edema, nausea, vomiting, and diarrhea [4]. Sensorineural hearing loss can result in up to one-third of LASV-infected patients even after recovery from the illness [5]. In fatal cases, patients may experience respiratory distress, encephalopathy, seizures, mucosal bleeding, shock, and coma [4].

The other prominent OW arenavirus is the lymphocytic choriomeningitis virus (LCMV), which is found worldwide and can cause severe diseases in immunocompromised individuals [6,7,8,9]. Approximately 5% of the human populations have been exposed to LCMV (based on available seroprevalence data) partly because its natural host (the house mouse) has worldwide distribution. Most acquired LCMV infections are non-symptomatic or mild [10,11], but congenital LCMV infection can be quite serious [12], resulting in spontaneous abortion and fetal death, or leaving the infant with neurological dysfunction. Because only serious congenital LCMV cases are reported, the true incidence of infection is not known [13]. LCMV infections in transplant recipients have also been shown to be quite serious. Eleven of fourteen known cases have proven to be fatal [7,8,9].

Among the NW arenaviruses, JUNV, Machupo (MACV), Guanarito (GTOV), Chapare (CHPV), and Sabiá (SABV) can cause HF with high mortality in humans (Table 1) [14,15,16,17,18,19,20,21,22]. Similar to HF caused by OW arenaviruses, patients infected with the NW viruses sometime experience fever, petechial hemorrhage, edema, respiratory distress, shock, thrombocytopenia, leukopenia, and mucosal bleeding. However, there are some differences between HF caused by NW or OW viruses. For example, whereas hepatitis is common in many severe LASV-infected cases, it is uncommon or mild in HF caused by the NW viruses. Additionally, neurologic symptoms, hemorrhaging, leukopenia, and thrombocytopenia seem to be more common in NW arenavirus-infected patients than in LASV-infected patients [23], the reasons for which are unknown.

## 2. New World (NW) and Old World (OW) Arenaviruses Trigger Different Immune Responses in Infected Hosts

Differential immune responses to NW and OW arenavirus infections appear to play an important role in their clearance. An effective T cell-mediated response appears to be critical for recovery from LASV infection. Experimentally infected macaques survived the infection partly due to the activated level of T cells, whereas fatal infections showed low and delayed T cell activation and high viral loads [24]. On the other hand, the levels of antibodies (e.g., IgG and IgM titers) were not associated with outcome of the disease in humans, indicating the that the antibody response is not effective in controlling LASV infection [25].

While the initial target cells of LASV infection may vary depending on the specific route of infection, antigen presenting cells (APCs)—such as dendritic cells (myeloid and/or stromal/mucosal DCs), monocytes, and macrophages—may be among early targets of LASV infections. In vitro, these cells are readily infected by LASV, but they fail to become activated [26] as evidenced by no increase in the levels of tumor necrosis factor alpha (TNFα) and interleukins (IL-1β, IL-6, or IL-12). Additionally, there is no increase in the levels of costimulatory molecules—such as CD80, CD86, CD40, CD54, or HLAs—which results in the absence of increased levels of phagocytic activity by the DCs. While data from LASV infected patients are limited, Mahanty and colleagues have compared the levels of a number of pro-inflammatory and anti-inflammatory cytokines and chemokines in serum samples collected from hospitalized patients with fatal and nonfatal LASV infections to those collected from uninfected control samples [27]. Their study showed that the levels of serum pro-inflammatory chemokines (IL-8 and interferon-inducible IP10 protein) were significantly higher in patients with acute nonfatal LASV infections than in control individuals. In sharp contrast, the levels of these chemokines were low or undetectable in patients with fatal LASV infections. Although the levels of TNFα were not increased in LASV-infected patients regardless of the disease status, the elevated levels of its receptors in the serum of infected patients suggested that TNFα might also play a role in the disease pathogenesis. IL-6 is highly upregulated in fatally infected NHPs and in LASV-infected humans, and elevated levels of IL-6 in plasma is currently being considered as a third biological marker of lethal Lassa fever in addition to viremia (>3.3 logs/mL) and liver enzyme aspartate aminotransferase (AST) (150 IU/mL) [25]. It is noteworthy that Branco and colleagues have recently analyzed the metabolic and immunological parameters of suspected LASV-infected patients in Sierra Leone, and have concluded that there is a strong correlation between survival and low levels of IL-6, IL-8, IL-10, macrophage inflammatory protein (MIP1β), CD40L, blood urea nitrogen (BUN), alkaline phosphatase (ALP), alanine aminotransferase (ALT), and AST [28].

Mopeia virus (MOPV) is closely related to LASV genetically, but it is nonpathogenic in humans and can actually provide protection against LASV infection in a nonhuman primate model [29]. Like LASV, MOPV infection also fails to activate DCs in culture [30] as evidenced by the failure to upregulate CD80, CD86, CD54, CD40, and HLA-abc. However, MOPV infection of cultured macrophages does increase the transcription of cellular genes encoding IFNα, IFNβ, TNF, and IL-6 [30]. Therefore, while MOPV fails to activate DCs, macrophages are capable of becoming activated when infected with this virus. A recent study has provided some tantalizing in vitro evidence to suggest that the L polymerase of MOPV can induce type I IFN production that is dependent on the RNA-dependent RNA polymerase activity of the L polymerase, the in vivo significance of which still needs to be demonstrated [31].

Unlike LASV-infected patients, those infected with the NW arenavirus JUNV showed elevated levels of cytokine expressions, such as TNFα, IFNα, IL-6, and IL-10 [32,33,34]. Although the increased levels of cytokines are observed in JUNV infected patients, in vitro infected macrophages and monocytes show no increase in cytokine production, such as IFNα, IFNβ, TNFα, IL-10, IL-6, and IL-12 [35]. Therefore, the increased cytokine levels observed in patients must originate from another source (e.g., DCs or other cell types). Recent studies have shown that human A549 lung epithelial cells infected with the NW JUNV or MACV can elicit an IFN response in culture [36,37]. In contrast to the OW LASV, both pathogenic NW JUNV and MACV can readily induce IFN production in cultured human primary DCs [36]. The exact role of cytokines in the pathogenesis of HF caused by NW arenaviruses has yet to be determined. A proposed theory is that cytokines may be important in controlling virus replication in the early stages of infection, while a delayed response could contribute to pathogenesis as seen in patients with severe disease and high levels of cytokines ([35] and reviewed in [38]).

The immune plasma from previously JUNV-infected patients seems to offer clinical benefits to those who are naively infected, especially early in the course of infection, which can reduce the mortality rate from 16% to 1% [39,40]. The antibody efficacy in the immune plasma to neutralize the virus leads to reduced viremia levels after plasma transfusion [41]. However, there are potential complications associated with plasma transfusion, such as a late neurologic syndrome that has been observed in 10% of treated patients [39]. Virus transmission from plasma transfusion is also a potential concern. While the high efficacy of immune plasma treatment of Argentine HF demonstrates the importance of an effective antibody response in JUNV-infected cases, immune plasma does not seem to play an important role in combating LASV infection. The reason for this disparity is unknown, but it underscores the differences in HF caused by OW and NW arenaviruses. A recent study has offered some tantalizing evidence for a potential role of the viral glycoproteins in evading humoral immune responses. It was demonstrated that the numbers of N-glycosylation sites on the arenaviral glycoproteins (i.e., GP1) was inversely correlated with the protective efficacy of neutralizing antibodies [42]. For example, JUNV contains less *N*-glycosylation sites on its GP1 than that of LASV, which appears to inversely correlate with the levels of neutralization offered by the antibodies generated by these proteins. Other examples of how some arenaviruses evade host immune recognition and activation are described in the next section.

## 3. Molecular Mechanisms of Immune Suppression by Pathogenic and Nonpathogenic NW and OW Arenaviruses

My laboratory as well as those of others have recently demonstrated that the LASV nucleoprotein (NP) serves as a potent inhibitor of type I IFNs by degrading immune stimulatory double-stranded RNAs (dsRNAs), thereby dampening both the innate and adaptive arms of cellular immunity [43,44]. While the source of the dsRNAs acting as pathogen-associated molecular patterns (PAMPs) in infected cells is unknown, they are thought to activate cellular sensor proteins, such as retinoic acid inducible gene 1 (RIG-I) or melanoma differentiation-associated protein 5 (MDA5), which in turn induces an antiviral signaling cascade [45,46]. Structural and biochemical analysis of several arenaviral NPs have revealed that these proteins share a highly conserved 3′–5′ DEDDh exoribonuclease domain at their C-termini [47,48] and that this exoribonuclease activity appears to mediate type I IFN suppression in vitro and in vivo [48,49,50]. Mutating any of the amino acids of the DEDDh active site of the NP exoribonuclease results in diminution or complete abrogation of the immunosuppressive function of NP. Recombinant viruses with DEDDh amino acid changes induce strong IFN expression and show a reduced growth potential in immune-competent cells and an attenuated phenotype in infected animals [43,49,51]. When these mutant viruses were serially passaged in immune-competent cells (e.g., human lung epithelial A549 cells) or used to infect animals (e.g., guinea pigs), the recovered viruses from cell culture or from many of the animals that succumbed to the infection showed evidence of reversion back to the wild-type NP sequence, emphasizing the strong genetic pressure to select for viruses with a functional NP 3′–5′ DEDDh exoribonuclease [48]. The NP proteins of OW LASV and LCMV as well as of the NW JUNV, MACV, Whitewater Arroyo virus (WWAV), Latino virus (LATV), and Tacaribe virus (TCRV) have all been shown to display the type I IFN suppressive function through the inhibition of IRF3 translocation [51,52,53] or by binding of the NP protein to IKKε in cell culture, thereby inhibiting the nuclear translocation and transcriptional activity of NFκB [54,55]. Therefore, it appears that this is a general mechanism of IFN suppression for both pathogenic and non-pathogenic NW and OW arenaviruses [53].

Besides NPs, some arenaviral Z proteins of NW arenaviruses (e.g., JUNV, GTOV, MACV, and SABV) have been shown to suppress type I IFN by directly binding to the cytosolic innate-immune sensor protein RIG-I, and resulting in downregulation of the IFNβ response [56]. My laboratory, in close collaboration with the Liang laboratory at the University of Minnesota, has recently demonstrated for the first time that the Z proteins from all known pathogenic arenaviruses—but not those of the non-pathogenic arenaviruses—can inhibit type I IFN by directly binding to the RIG-I and MDA-5 proteins and that the interactions interfere with the binding of these proteins to a downstream signaling protein MAVS, thus preventing the production of type I IFN [57]. While we have not determined the atomic structure(s) of the viral Z and RIG-I or MDA5 interactions, we have mapped the interactive domains to the N terminus (roughly 30 amino acids) of the Z proteins and the two CARD domains of RIG-I and MDA5. Exactly how the N-termini of the Z proteins from the pathogenic viruses interact with the CARD domains of RIG-I and MDA5 remains to be determined. We showed that cultured primary human macrophages, upon infection with some representative pathogenic arenaviruses, failed to elevate the levels of activating markers CD80, CD86 and of cytokines (e.g., IFNβ, TNFα, IL-1β, IL-6, and IL-8) [58]. We also showed that the infected human primary macrophages in vitro failed to mature as evidenced by the absence of increased levels of phagocytic activity. The failure of these cells to be activated upon pathogenic arenaviral infection leads to the lack of INF-γ production in a macrophage-T cells and macrophage-NK cells co-culture systems [58].

## 4. Vaccine Development for the NW (JUNV) and OW (LASV) Arenaviruses

Currently, no licensed vaccines are available for the prevention of LASV infection. Injection of LASV that has been inactivated by γ irradiation can generate good humoral responses in nonhuman primates, but this response fails to protect animals against lethal challenge with LASV [59], confirming the need for cell-mediated immunity against LASV [60]. It has been reported that patients who recovered from acute LASV infection were not associated with the presence of either IgG or IgM [25]. However, recent analysis of survivors of suspected LASV infections in Sierra Leone suggests that LASV-specific IgM seem to persist for months to years after initial infection [28]. However, positive LASV IgM seropositivity was determined not to be a reliable biomarker of acute Lassa fever based on the adjusted odds ratio of the LASV antigen-positive and IgM-positive versus the antigen-positive and IgM-negative individuals. The same group of investigators has also provided some tantalizing evidence to suggest that a soluble form of the GP1 subunit (sGP1) of the viral glycoprotein can be detected in the sera of a small number of the suspected cases (2/46), the significance of which is unknown [61]. Evidence for an important role of CD4+ T cells in protection comes from an experiment in which mice were vaccinated with recombinant vaccinia virus expressing the LASV glycoprotein precursor complex (GPC) that conferred cross-protection against LCMV via a CD4+ T-cell clone specific for the LASV GPC [62]. Ter Meulen and colleagues have further shown that lymphocytes of a LASV-seropositive individual can give rise to several CD4+ T-cell clones that can produce high levels of IFNγ upon stimulation with recombinant GP2 subunit of the viral glycoprotein GPC [63]. The same group of investigators has also shown that sera collected from LASV-seropostive individuals have very strong memory CD4+ T-cell responses against NP [64]. A recently published study also showed that CD4+ and CD8+ T cells can drive the disease phenotype (i.e., fatal meningoencephalitis) in the TCRV-infected mouse model [65]. By transplanting wild-type bone-marrow cells into irradiated type I IFN receptor knockout mice (*IFNAR*^−/−^), Oestereich and colleagues have shown that this new mouse model can recapitulate some important features of severe Lassa fever in humans, including T cell-mediated immunopathology [66]. It is therefore possible that while T cells are necessary for clearance of the virus, these cells may also be responsible for the disease if the host immune response proves incapable of clearing the virus infection [60].

As previously mentioned, MOPV infection had been shown to be capable of providing protective immunity against LASV infection in nonhuman primates [29]. The recombinant ML29 vaccine that carries in its genome the large (L) genomic segment of the MOPV and the small (S) genomic segment of the LASV has been shown to protect marmosets against LASV infection by inducing sterilizing cell-mediated immunity [67]. Recombinant vaccinia virus has also been used to develop LASV vaccine that expresses combinations of LASV NP and the GP1 and GP2 glycoprotein subunits. Those recombinant vaccinia viruses that express all three proteins—or a combination of the GP1 and GP2 glycoproteins—have also been shown to be able to protect macaques from fatal infection but not viremia. Preexisting immunity from prior smallpox vaccination, however, would potentially attenuate the protective immunity of this vaccinia-based LASV vaccine [29]. A recombinant VSV vaccine expressing the LASV GPC has also been developed and found to be protective in nonhuman primates against LASV infection. However, LASV viremia levels were detected in the animals at day 7 post challenge [68]. The YF17D yellow-fever virus (YFV) vaccine vector has also been used to express the LASV glycoprotein. A virus construct that contains both glycoproteins from YFV and LASV has been shown to be capable of protecting inbred strain 13 guinea pigs from LASV infection. However, this YFV-based LASV vaccine replicates poorly and is not stable in cell culture [69,70]. Another YFV construct expressing only LASV GP has also been shown to protect strain 13 animals from death, but it does not prevent disease or viremia and has been found to be poorly immunogenic [70].

Unlike vaccine development for LASV, the JUNV vaccine development effort has been more fruitful. The Candid #1 strain of JUNV, developed jointly by the U.S. Army Medical Research Institute of Infectious Diseases (USAMRIID) and the Argentine Ministry of Health, was generated by passaging the virus twice in guinea pigs, followed by sequential passaging in brain of suckling mice and in cultured fetal rhesus monkey lung cells [71]. It is a highly successful vaccine against Argentine HF with an estimated effectiveness of 98.1%. Unfortunately, it is not an appealing vaccine candidate for commercial production because of its small target population (100–1000 cases per year). That being said, Candid #1 is currently being used in Argentina as an effective vaccine against JUNV infection, effectively reducing annual infection from 300–1000 to mere 30–50 cases (Table 1). However, this vaccine has not been approved by the US Food and Drug Administration (FDA) due partly to a lack of proper FDA compliant documentation, the lack of detailed genetic composition and molecular mechanism of attenuation of the vaccine strain, and the association of foot-and-mouth disease in several regions of Argentina. A reverse genetics system has recently been developed for Candid #1, which has the potential to address all of these concerns, and hopefully provide a safe and effective vaccine which would meet FDA approval criteria [72].

## 5. Conclusions

Arenaviruses are significant human pathogens. While the OW and NW arenaviruses share many characteristics, differences do exist when it comes to how the infected hosts respond to their infections immunologically. While an effective T cell mediated response is critical for the clearance of LASV infection, the antibody response seems to be important for recovery from JUNV infection, as evidenced by the efficacy of treatment with immune plasma. While severe LASV infection is characterized by a generalized immune suppression, JUNV infection seems to result in a cytokine storm. A better level of understanding of the differences between these closely related viruses will aid the development of new vaccines and treatment methods against these deadly viral pathogens. Due to various reasons, the development of vaccines against OW and NW arenaviruses has been slow and challenging. That being said, recent scientific progress coupled with a renewed international interest and investment in vaccine development for several highly pathogenic yet often ignored viruses by the Coalition for Epidemic Preparedness Innovations (CEPI) [73,74] has generated a lot of excitement and optimism toward the development of new vaccines for some of these deadly human viral pathogens, including LASV.

## Figures and Tables

**Table 1:** Old World (OW) and New World (NW) arenaviruses.

Classification	Virus	Geographic Location	Incidence of Disease
Old World	LCMV	Worldwide	Over 5% of people show evidence of prior exposure, <1% mortality
	Lassa virus	West Africa	Up to 500,000 infections annually, ~5000 deaths/year
	Lujo virus	South Africa	5 identified cases, 4 fatal
New World	Junín virus	Argentina	300–1000 cases/year before Candid #1 vaccination program, 30–50 cases/year after introduction of vaccine, 15–30% mortality
	Machupo virus	Bolivia	1962–1964: 1000 cases; 1990s: 19 cases; 2007–2008: >200 cases; ~20% mortality
	Sabiá virus	Brazil	1 naturally occurring case, fatal
	Guanarito virus	Venezuela	618 cases, 23% fatal
	Chapare virus	Bolivia	1 confirmed case, fatal
